# Feasibility and Safety of Cerebral Embolic Protection Device Insertion in Bovine Aortic Arch Anatomy

**DOI:** 10.3390/jcm9124118

**Published:** 2020-12-20

**Authors:** Ana Paula Tagliari, Enrico Ferrari, Philipp K. Haager, Martin Oliver Schmiady, Luca Vicentini, Mara Gavazzoni, Marco Gennari, Lucas Jörg, Ahmed Aziz Khattab, Stefan Blöchlinger, Francesco Maisano, Maurizio Taramasso

**Affiliations:** 1 Cardiac Surgery Department, University Hospital of Zurich, University of Zurich, 8091 Zurich, Switzerland; enrico.ferrari@cardiocentro.org (E.F.); philipp.haager@kssg.ch (P.K.H.); martinoliver.schmiady@usz.ch (M.O.S.); luca.vicentini@usz.ch (L.V.); mara.gavazzoni@usz.ch (M.G.); marco.gennari@usz.ch (M.G.); lucas.joerg@kssg.ch (L.J.); francesco.maisano@usz.ch (F.M.); m.taramasso@gmail.com (M.T.); 2 Postgraduate Program in Health Sciences: Cardiology and Cardiovascular Sciences—Faculdade de Medicina, Universidade Federal do Rio Grande do Sul, Porto Alegre 90035003, Brazil; 3 Cardiac Surgery Department, Cardiocentro Ticino, 6900 Lugano, Switzerland; 4 Cardiology Department, Kantonsspital St. Gallen, 9007 St. Gallen, Switzerland; 5 Cardiac Surgery Department, IRCCS Centro Cardiologico Monzino, 20138 Milan, Italy; 6 Cardiology Department, University Hospital of Zurich, University of Zurich, 8091 Zurich, Switzerland; ahmedaziz.khattab@usz.ch; 7 Cardiology Department, Cardiance Clinic, 8808 Pfäffikon, Switzerland; 8 Cardiology Department, Kantonsspital Winterthur KSW, 8400 Winterthur, Switzerland; stefan.bloechlinger@ksw.ch

**Keywords:** cerebral protection device, transcatheter aortic valve replacement, stroke, cerebrovascular events, bovine aortic arch

## Abstract

Background: Cerebral embolic protection devices (CEPDs) have emerged as a mechanical barrier to prevent debris from reaching the cerebral vasculature, potentially reducing stroke incidence. Bovine aortic arch (BAA) is the most common arch variant and represents challenge anatomy for CEPD insertion during transcatheter aortic valve replacement (TAVR). Methods: Cohort study reporting the Sentinel^TM^ Cerebral Protection System insertion’s feasibility and safety in 165 adult patients submitted to a transfemoral TAVR procedure from April 2019 to April 2020. Patients were divided into 2 groups: (1) BAA; (2) non-BAA. Results: Median age, EuroScore II, and STS score were 79 years (74–84), 2.9% (1.7–6.2), and 2.2% (1.6–3.2), respectively. BAA was present in 12% of cases. Successful two-filter insertion was 86.6% (89% non-BAA vs. 65% BAA; *p* = 0.002), and debris was captured in 95% (94% non-BAA vs. 95% BAA; *p* = 0.594). No procedural or vascular complications associated with Sentinel insertion and no intraprocedural strokes were reported. There were two postprocedural non-disabling strokes, both in non-BAA. Conclusion: This study demonstrated Sentinel insertion feasibility and safety in BAA. No procedural and access complications related to Sentinel deployment were reported. Being aware of the bovine arch prevalence and having the techniques to navigate through it allows operators to successfully use CEPDs in this anatomy.

## 1. Introduction

Although newer-generation transcatheter heart valve devices and increased operator experience have reduced the incidence of cerebrovascular events during transcatheter aortic valve replacement (TAVR) [1,2], stroke remains one of the most feared procedural complications. This concern is especially relevant since TAVR is moving to low-risk and younger patients, a population in which a cerebrovascular event has even more impact on survival and quality of life [3,4,5,6].

Cerebral embolic protection devices (CEPDs) have been developed to work as a mechanical barrier to prevent embolic debris from reaching the cerebral vasculature, potentially reducing neurological events during TAVR procedures. The dual-filter-based Sentinel^TM^ Cerebral Protection System (Sentinel) (Boston Scientific, Marlborough, MA, USA) received CE Mark approval in 2013 and Food and Drug Administration (FDA) approval in 2017, and it is now the most widely used CEPD system [7,8].

Although no single study had demonstrated Sentinel benefits in terms of hard outcomes, two recently published propensity scoring match analyses have suggested that Sentinel use was associated with reduced post-procedural stroke and mortality rates. In the Society of Thoracic Surgeons/American College of Cardiology Transcatheter Valve Therapy (STS/ACC TVT) Registry, after propensity-weighted analysis, significant reduction in in-hospital stroke [relative risk (RR) 0.82; 95% confidence interval (CI) 0.69-0.97], in-hospital death or stroke (RR 0.84; 95% CI 0.73-0.98), 30-day stroke (RR 0.85; 95% CI 0.73-0.99), and 30-day mortality rate (RR 0.78; 95% CI 0.64-0.95) was observed in patients submitted to a protected TAVR [9]. Corroborating these findings, another propensity-weighted analysis from the National Inpatient Sample showed that Sentinel use was associated with lower risk of in-hospital ischemic stroke [odds ratio (OR) 0.24; 95% CI 0.09-0.62] and in-hospital death (0 vs. 1%; *p* = 0.036) [10].

Bovine aortic arch is the most common aortic arch variant and occurs when the brachiocephalic artery (or innominate artery) shares a common origin with the left common carotid artery. The bovine aortic arch prevalence is around 15% (range from 8% to 25%) [11], and its presence carries important implications for preprocedural planning and open or endovascular interventions involving the aortic arch. Indeed, the bovine arch has been associated with consistent geometric hostile features for endovascular procedures, namely angulation, tortuosity, and elongation [12]. Bovine arch is also a recognized anatomic risk factor for carotid stenting, increasing the procedural difficulty level [13], and thoracic aortic disease development [14]. In this respect, in younger patients with this anatomical configuration, TAVR may represent a valid option considering that they could, in time, require an open aortic valve repair.

Regarding CEPD insertion in bovine aortic arches, though there is no formal contraindication to apply the Sentinel system in this scenario, the angulation and tortuosity features related to this anatomical variant are frequent reasons to preclude Sentinel use in real-life procedures. Therefore, many patients who could benefit from cerebral protection are deprived of this strategy.

Herein, we report the feasibility and safety of Sentinel insertion in bovine aortic arch anatomy and bovine arch prevalence in patients undergoing a TAVR procedure. This is the first study evaluating a cohort of patients with bovine aortic arch anatomy submitted to TAVR under cerebral protection. 

## 2. Material and Methods

Single-center cohort study. Patients who underwent a transfemoral-protected TAVR from April 2019 to April 2020 were analyzed and divided into two groups according to the aortic arch anatomy: Group 1: Non-bovine aortic arch anatomy; Group 2: Bovine aortic arch anatomy.

All procedures involving human participants followed the institutional research committee ethical standards in accordance with the 1964 Helsinki declaration and its later amendments. TAVR indication decisions were driven by the institutional heart team, and patients provided written informed consent before the procedure. Patients undergoing TAVR procedures in our institution are included in the nationwide Swiss TAVI Registry (NCT01368250; 2016-00587), a prospective multi-center and observational national registry collecting clinical characteristics of patients undergoing TAVR in Switzerland, which had been previously approved by local ethics committees [15,16]. 

Clinical, echocardiographic, and tomographic data were collected at baseline, discharge, and 30 days after the procedure. Clinical events were adjudicated according to the updated Valve Academic Research Consortium (VARC-2) criteria [17]. Combined procedures were defined as simultaneous elective interventions, such as coronary artery angiogram, percutaneous coronary artery intervention, left atrial appendage occlusion, intravascular lithotripsy, bioprosthetic or native aortic scallop intentional laceration to prevent coronary artery obstruction (BASILICA), or pacemaker generator change. Significant tortuosity was defined, based on subjective operator judgment, as a brachiocephalic or left common carotid artery S- or C-shaped elongation or undulation, evaluated in the preoperative computed tomography (CT) scan.

**Figure 1:**
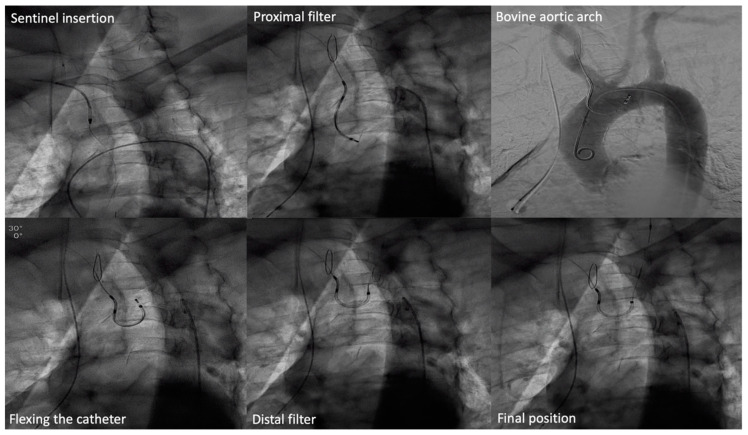
Sentinel insertion in a bovine aortic arch anatomy.

The cerebral embolic protection device used was the dual-filter-based Sentinel^TM^ Cerebral Protection System (Sentinel) (Boston Scientific, Marlborough, MA, USA), which consists of a 6-Fr-compatible steerable catheter (100 cm long) carrying two cone-shaped, biocompatible polyurethane filters equipped with 140 μm pores to capture and retrieve debris during TAVR procedures. The sheath is inserted through the right radial artery, and the filters are targeted to the brachiocephalic artery (proximal target vessel) and the left common carotid artery (distal target vessel). Using an articulating sheath, the device’s curve can be adjusted to accommodate anatomic variations of the aortic arch (Figure 1, Movie 1). In patients in whom the insertion of both filters was not possible, only the proximal filter was deployed. At the end of the procedure, both filters were checked for the presence of captured material. Successful Sentinel insertion was defined as a successful positioning and deployment of both filters in the correct anatomical position.

## 3. Statistical Analysis

Quantitative data were expressed as mean ± standard deviation (SD) or median and interquartile range (IQR). Qualitative variables were expressed as frequency and percentage. Analyses were performed using the statistical package SPSS 19.0 software (Chicago, IL, USA). Categorical variables were analyzed using the chi-square test, continuous variables were analyzed using the Student’s T-test or the Mann–Whitney U test. A two-sided *p*-value lower than 0.05 was considered significant for all tests.

## 4. Results

From April 2019 to April 2020, 231 patients were submitted to a transfemoral TAVR procedure, 165 (71.5%) of them under cerebral embolic protection. The most common reasons to preclude Sentinel use were significant aortic arch branch tortuosity (22.3%, *n* = 15); emergency procedure or procedure performed under hemodynamic instability (10.4%, *n* = 7); no right radial artery suitable for Sentinel insertion (9%, *n* = 6) or no Sentinel progression (3%, *n* = 2); aberrant right subclavian artery (3%, *n* = 2); and previous left carotid endarterectomy (3%, *n* = 2). 

**Figure 2:**
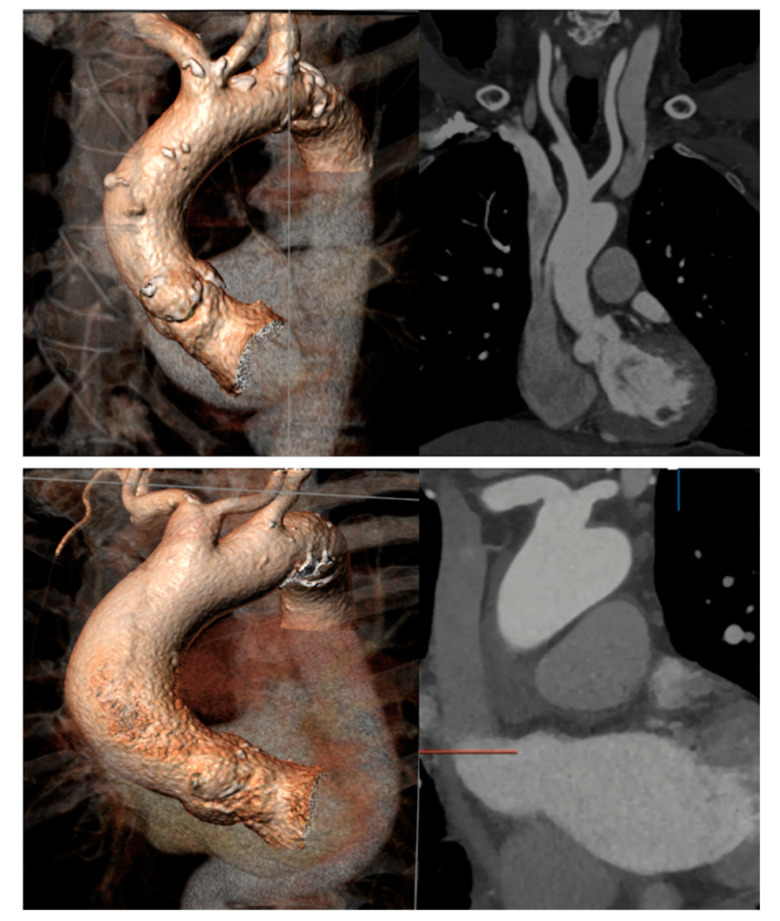
Two examples of bovine aortic arch anatomy suitable for Sentinel insertion.

Overall, bovine aortic arch (Figure 2) was identified in 37 patients (16%, *n* = 37/231) and in 20 (12.12%; *n* = 20/165) of those submitted to a protected TAVR procedure. Type I (common origin of the brachiocephalic and left common carotid artery) bovine arch anatomy was presented in 97.3% (*n* = 36) of the cases, and type II (left common carotid artery originating directly from the brachiocephalic artery, rather than as a common trunk) in 2.7% (*n* = 1). Comparison between patients who received a Sentinel device with those who did not are presented in the Supplementary Material (Table S1). There was no difference in procedural time (55 min (46–67) vs. 51.5 min (41.7–62.7); *p* = 0.492) or injected contrast volume (87 mL (69–133) vs. 102 (77–120); *p* = 0.071) between protected and unprotected TAVR. 

Among the 165 patients who underwent a transfemoral TAVR under cerebral protection, baseline clinical and aortic valve characteristics were similar between the bovine and non-bovine anatomy groups and are presented in Table 1. Significant aortic arch branch tortuosity was present in 27 patients (16.3%; 17.2% in non-bovine vs. 2% in bovine; *p* = 0.412). Successful insertion of two Sentinel filters was achieved in 143 (86.6%; 89.7% in non-bovine vs. 65% in bovine; *p* = 0.002). Debris was captured in the filters of 158 patients (95.7%; 94.5% in non-bovine vs. 95% in bovine; *p* = 0.594). 

Procedure characteristics and outcomes are presented in Table 2 and Table 3, respectively. There were no procedural or vascular complications associated with Sentinel insertion, nor intraprocedural strokes. Two non-disabling ischemic strokes (1.21%) were reported in the non-bovine group: the first case showed-up as aphasia on the first postoperative day, which completely regressed one day after; the second case presented hemiplegia on the third postoperative day, which also totally regressed at the hospital discharge. No new cerebrovascular events were reported between hospital discharge and 30-day outpatient evaluation. Total procedure time (55 min vs. 55 min; *p* = 0.654) and volume of contrast used (87mL vs. 89mL; *p* = 0.727) were similar in bovine and non-bovine aortic arches, respectively.

## 5. Discussion

Cerebrovascular events are one of the most devastating TAVR complications, not only in terms of mortality but also regarding the potential sequelae and impaired quality of life [3,4,5,6]. Clinical strokes are related to an up-to-nine-fold increase in postprocedural mortality [4,18,19], non-return to working life in 50% of the cases [20,21], and an increase in index hospitalization cost of approximately 25,000 USD [22].

Almost 50% of all early post-TAVR strokes are directly procedure-related and occur within the first 24 h [3,19,23]. This post-TAVR stroke incidence peak is consistent with what has been observed in carotid stenting procedures, suggesting that stroke occurrence is related to hostile aortic arch and anatomical features of supra-aortic vessels [24]. 

CEPDs were developed with the purpose of offering a safer procedure, mitigating cerebrovascular event risk, and improving TAVR-related outcomes [25,26,27,28,29]. Despite the worldwide spread of CEPD use, evidence about anatomical features associated with its unsuccessful implantation remains scarce [29]. As bovine aortic arch is the most common aortic arch branching variant in humans, the present study aimed to report the feasibility and safety of performing a Sentinel device insertion in this anatomy, as well as the prevalence of bovine aortic arch anatomy in patients who underwent a protected TAVR. 

Previous studies have indicated that bovine left common carotid artery configuration occurs in 8–25% of patients [11], a prevalence similar to that observed in our cohort (12%; *n* = 20/165). The presence of this type of anatomical configuration is associated with an increased endovascular device navigation complexity [30,31]. Comparing patients with or without aortic arch anomalies who underwent a carotid artery stent, Faggioli et al. observed that bovine arch was associated with increased neurologic events (20% vs. 5.3%; *p* = 0.039) and technical failure (89.6% vs. 76.4%; *p* = 0.1) due to the greater difficulty in navigating devices through tortuous vessels [30]. In addition, the presence of increased aortic arch angulation also reflects a hostile take-off angle of the supra-aortic branches [12]. In this scenario, Rozado et al. advocated that an extreme device tip flexure could help to advance a wire into the left carotid artery, allowing proper Sentinel advancement and positioning [32].

In our study, despite bovine aortic arch anatomy being associated with reduced two-filter insertion (89.7% vs. 65%; *p* = 0.002), this feature did not reflect an increase in procedural complication rate or postprocedural neurological events. Total procedure time (55 min vs. 55 min; *p* = 0.654) and volume of contrast used (87 mL vs. 89 mL; *p* = 0.727) were also similar in bovine and non-bovine aortic arches. Higher tortuosity degree and challenging device navigation were probably factors related to a lower rate of two-filter insertion in bovine group. However, since in bovine aortic arches, both common carotid arteries have the same origin and are in a close position, one filter properly positioned beyond their origins is probably enough to provide adequate cerebral protection. Furthermore, even if bi-carotid protection is not feasible, a single-filter insertion is possibly better than no cerebral protection at all. Indeed, further computational fluid dynamics studies may shed some light on stroke risk related to debris distribution along the arch and supra-aortic branches according to the aortic arch anatomy.

In our study, the Sentinel was not used in 28.5% (*n* = 66) of patients, a rate similar to that recently reported by Voss et al. (38.5%; *n* = 122). In this study, the authors reported that Sentinel ineligibility reasons, based on MSCT criteria, were as follows: inappropriate diameter within the target landing zone (*n* = 116); significant subclavian artery stenosis (*n* = 4) or an aberrant subclavian artery (*n* = 3); and clinical characteristics including hypersensitivity to nickel titanium (*n* = 1), radial artery occlusion (*n* = 1), or previous left common carotid artery interventions (*n* = 5) [33]. 

Another important anatomic consideration concerning Sentinel insertion eligibility is the presence of vascular tortuosity. Tortuosity hampers access to the filter-landing zone [34,35,36], increasing device manipulation, contrast use, vessel injury risk, and CEPD insertion failure [35]. Device instructions stipulate that Sentinel should be avoided in patients with “excessive” vessel tortuosity; however, there is no specific definition of what excessive tortuosity means. In our study, the overall prevalence of aortic arch branches tortuosity was 16.4% (*n* = 27/165), with no significant difference in tortuosity distribution between bovine and non-bovine Sentinel groups (17.2% in non-bovine vs. 2% in bovine; *p* = 0.412). 

Considering the benefits of cerebral protection during TAVR, even though no randomized trial had found significant stroke or mortality reduction, a propensity-matched cohort study by Seeger et al. identified lower mortality or all-stroke rate 7 days post-TAVR when a CEPD was used (2.1% vs. 6.8%; *p* = 0.01). All-stroke rate was also inferior in protected TAVR (1.4% vs. 4.6%, *p* = 0.03; OR 0.29, 95% CI 0.10-0.93; NNT 31). In multivariable analysis, STS score (*p* = 0.02) and TAVR without cerebral protection device (*p* = 0.02) were independent predictors for the primary endpoint (mortality or stroke) [37]. Two years after this initial study, the same authors evaluated the incidence of procedural stroke within 72 h post-TAVR in a propensity-matched population comprising patients from the SENTINEL US IDE trial [24], the CLEAN-TAVI trial [34], and SENTINEL-Ulm registry (University Hospital of Ulm, Ulm, Germany) (*n* = 1306). The main result showed that the procedural all-stroke rate was significantly lower in the CEPD group compared to the unprotected group (1.88% vs. 5.44%; OR 0.35, 95% CI 0.17-0.72). In addition, the combined outcome of all-cause mortality and all-stroke was significantly lower (2.06% vs. 6.00%; OR 0.34, 95% CI 0.17-0.68) in the protected group [38]. These findings were supported by two recently released propensity scoring match analyses showing benefit in terms of stroke and mortality rate reduction when Sentinel was used [9,10]. 

Regarding Sentinel’s cost-effectiveness, estimations show that the cost of preventing a single stroke or death is around 60,000 USD [39]. As the Sentinel device costs approximately 2800 USD, according to Giustino et al., a total amount of 61,600 USD should be spent to prevent one stroke or death. This value seems to be justifiable given the negative physical, emotional, and economic impact of stroke [40].

## 6. Limitations

The present analysis reflects a single-center, non-randomized, but prospectively acquired experience. Therefore, all the inherent limitations of such design need to be taken into account. In addition, our results are based on a single specific cerebral embolic protection device and cannot be generalized to other available devices. Despite our small sample size, this report represents the first cohort of patients with bovine aortic arch anatomy successfully treated with TAVR procedure under cerebral protection.

## 7. Conclusions

This study demonstrated Sentinel insertion feasibility and safety in bovine aortic arch anatomy. No procedural and access complications related to Sentinel deployment were reported. Being aware of the bovine arch prevalence and having the techniques to navigate through it allows operators to successfully use Sentinel in this anatomy.

## Figures and Tables

**Table 1:** Baseline clinical and aortic valve characteristics in patients undergoing transcatheter aortic valve replacement (TAVR) with concomitant cerebral protection.

Variable	Non-Bovine *n* = 145	Bovine *n* = 20	*p*-Value
Age, years median (IQR)	79 (74–83)	80 (77–84)	0.318
Male gender	86 (59.3)	14 (70)	0.359
EuroScore II, % median (IQR)	2.8 (1.6–6.2)	3.2 (2.2–6.3)	0.328
STS score, % median (IQR)	2.1 (1.6–3.2)	2.8 (1.6–3.7)	0.732
Weight, Kg mean ± SD	77.2 ± 14	75.9 ± 16	0.717
Height, cm mean ± SD	166.4 ± 8	170 ± 10	0.051
Severe aortic valve stenosis	142 (97.9)	20 (100)	0.516
Aortic valve regurgitation ≥ moderate	11 (6.6)	1 (5)	0.561
NYHA functional class III/IV	77 (53)	11 (55)	0.982
Arterial hypertension	103 (71)	13 (65)	0.580
Diabetes mellitus	41 (28.3)	2 (10)	0.081
Dyslipidemia	84 (57.9)	12 (60)	0.182
Coronary artery disease	64 (44.1)	12 (60)	0.191
Previous myocardial infarction	17 (12.4)	4 (20)	0.349
Previous stroke	11 (7.6)	3 (15)	0.265
Atrial fibrillation	50 (34.5)	11 (55)	0.075
Chronic obstructive pulmonary disease	17 (11.7)	3 (15)	0.674
Chronic kidney disease	44 (30.3)	6 (30)	0.975
Anemia	16 (11)	0	0.118
Peripheral artery disease	12 (8.3)	1 (5)	0.610
Active smoker	46 (31.7)	8 (40)	0.460
Previous PCI	37 (25.5)	9 (45)	0.069
Previous CABG	8 (5.5)	3 (15)	0.111
Previous aortic valve surgery	9 (6.2)	1 (5)	0.832
Previous permanent pacemaker	11 (7.6)	2 (10)	0.707
Bicuspid aortic valve	14 (9.7)	1 (5)	0.497
Aortic valve area, cm^2^ median (IQR)	0.75 (0.6–0.9)	0.85 (0.7–0.97)	0.099
Aortic valve mean gradient, mmHg median (IQR)	42 (35–51)	45 (37–52)	0.703
LVEF, % median (IQR)	58 (45–65)	55 (47–60)	0.301

Values expressed as numbers (%) unless otherwise indicated. IQR = interquartile range; SD = standard deviation; CABG = coronary artery bypass graft; LVEF = left ventricular ejection fraction; NYHA = New York Heart Association; PCI = percutaneous coronary intervention; STS = The Society of Thoracic Surgeons.

**Table 2:** Procedural characteristics.

Variable		Non-Bovine *n* = 145	Bovine *n* = 20	*p*-Value
Sedation		136 (94.4)	19 (95)	0.959
Combined procedure		9 (6.2)	0	0.252
Two Sentinel filters inserted		130 (89.7)	13 (65)	0.002
Type of bioprosthesis				0.908
	Portico Edwards Sapien 3/Ultra Medtronic Evolut R/Pro Acurate Neo Allegra Lotus	49 (33.8) 43 (29.6) 36 (24.8) 12 (8.3) 3 (2.1) 2 (1.4)	9 (45) 5 (25) 3 (15) 2 (10) 1 (5) 0	
Procedure time, min median (IQR)		55 (45–67)	55 (48–61)	0.654
Contrast injection, mL median (IQR)		87 (68–130)	89 (72–145)	0.727

Values expressed as numbers (%) unless otherwise indicated. IQR = interquartile range.

**Table 3:** In-hospital outcomes.

Variable	Non-Bovine *n* = 145	Bovine *n* = 20	*p*-Value
All-cause mortality	1 (0.7)	0	0.710
Permanent pacemaker implantation	20 (13.8)	5 (25)	0.190
Non-disabling stroke	2 (1.3)	0	0.516
New onset of atrial fibrillation	6 (4.1)	0	0.354
Delirium	3 (2.1)	0	0.516
Aortic valve mean gradient, mmHg median (IQR)	8.8 (5–11)	7.7 (5–9)	0.309
Aortic valve regurgitation ≤ mild	135 (93.1)	18 (90)	0.909
LVEF, % median (IQR)	57 (49–63)	54 (49–57)	0.214
Hospital length of stay, days median (IQR)	5 (4–7)	6 (4–7)	0.554

Values expressed as numbers (%) unless otherwise indicated. IQR = interquartile range; LVEF = left ventricular ejection fraction.

## Supplementary Materials

The following are available online at https://www.mdpi.com/2077-0383/9/12/4118/s1, Table S1: Baseline clinical and aortic valve characteristics in patients who received or not a Sentinel device during a transfemoral TAVR procedure; Movie S1: Sentinel cerebral protection device implanted in a bovine aortic arch anatomy.

## Abbreviations

Bioprosthetic or native aortic scallop intentional laceration to prevent coronary artery obstruction (BASILICA); bovine aortic arch (BAA); cerebral embolic protection device (CEPD); confidence interval (CI); dual-filter-based Sentinel^TM^ Cerebral Protection System (Sentinel); Food and Drug Administration (FDA); interquartile range (IQR); multislice computed tomography (MSCT); odds ratio (OR); relative risk (RR); standard deviation (SD); Society of Thoracic Surgeons/American College of Cardiology (STS/ACC); transcatheter aortic valve replacement (TAVR); The Society of Thoracic Surgeons (STS); Valve Academic Research Consortium (VARC-2).
