# Supplementary material for: Feasibility and Safety of Cerebral Embolic Protection Device Insertion in Bovine Aortic Arch Anatomy
Source: J Clin Med. 2020 Dec 20;9(12):4118. doi: 10.3390/jcm9124118 (PMC7766100; doi:10.3390/jcm9124118)
Supplement: Supplementary file 1 [file jcm-09-04118-s001.zip › jcm-1026264-Supplementary table S1.docx]

**Table S1.** Baseline clinical and aortic valve characteristics in patients who received or not a Sentinel device during a transfemoral TAVR procedure.

| **Variable** | **Non-Sentinel**  **n = 66** | **Sentinel**  **n = 165** | ***p*-value** |
| --- | --- | --- | --- |
| Age, years median (IQR) | 78.5 (74-86) | 79 (74-84) | 0.950 |
| Male gender | 46 (69.7) | 100 (60.6) | 0.196 |
| EuroScore II, % median (IQR) | 3.4 (2.1-5.8) | 2.9 (1.7-6.2) | 0.282 |
| STS score, % median (IQR) | 2.6 (1.5-3.7) | 2.2 (1.6-3.2) | 0.163 |
| Weight, kg mean ± SD | 74.8 ± 14 | 77 ± 15 | 0.294 |
| Height, cm mean ± SD | 166 ± 9.7 | 167 ± 8.6 | 0.443 |
| Bovine aortic arch | 17 (25.8) | 20 (12.1) | 0.011 |
| Aortic arch branches tortuosity | 15 (22.7) | 27 (16.4) | 0.257 |
| Severe aortic valve stenosis | 64 (97) | 162 (98.2) | 0.567 |
| Aortic valve regurgitation ≥ moderate | 9 (13.6) | 12 (7.3) | 0.323 |
| NYHA functional class III/IV | 33 (50) | 88 (52.3) | 0.322 |
| Arterial hypertension | 49 (74.2) | 116 (70.3) | 0.549 |
| Diabetes mellitus | 12 (18.2) | 43 (26.1) | 0.204 |
| Dyslipidemia | 29 (43.9) | 96 (58.2) | 0.05 |
| Coronary artery disease | 31 (47) | 76 (46.1) | 0.9 |
| Previous myocardial infarction | 8 (12.1) | 22 (13.3) | 0.804 |
| Previous stroke | 7 (10.6) | 14 (8.5) | 0.612 |
| Atrial fibrillation | 24 (36.4) | 61 (37) | 0.931 |
| Chronic obstructive pulmonary disease | 12 (18.2) | 50 (30.3) | 0.078 |
| Chronic kidney disease | 28 (42.4) | 6 (30) | 0.975 |
| Peripheral artery disease | 6 (9.1) | 13 (7.9) | 0.762 |
| Active smoker | 19 (28.8) | 54 (32.7) | 0.561 |
| Previous PCI | 17 (25.8) | 46 (45) | 0.744 |
| Previous CABG | 10 (15.2) | 11 (6.7) | 0.043 |
| Previous aortic valve surgery | 1 (1.5) | 10 (6.1) | 0.143 |
| Previous permanent pacemaker implantation | 4 (6.1) | 13 (7.9) | 0.633 |
| Bicuspid aortic valve | 2 (3) | 15 (9.1) | 0.111 |
| Aortic valve area, cm^2^ median (IQR) | 0.8 (0.7–0.9) | 0.8 (0.6–0.9) | 0.720 |
| Aortic valve gradient, mmHg median (IQR) | 42 (32–52) | 42 (35–51) | 0.894 |
| LVEF, % median (IQR) | 57 (48–63) | 57 (45–65) | 0.970 |

Values expressed as numbers (%) unless otherwise indicated. IQR = interquartile range; CABG = coronary artery bypass graft; LVEF = left ventricular ejection fraction; SD = standard deviation; NYHA = New York Heart Association; PCI = percutaneous coronary intervention; SD = standard deviation.
